# Interreality for the management and training of psychological stress: study protocol for a randomized controlled trial

**DOI:** 10.1186/1745-6215-14-191

**Published:** 2013-06-28

**Authors:** Federica Pallavicini, Andrea Gaggioli, Simona Raspelli, Pietro Cipresso, Silvia Serino, Cinzia Vigna, Alessandra Grassi, Luca Morganti, Margherita Baruffi, Brenda Wiederhold, Giuseppe Riva

**Affiliations:** 1 Applied Technology for Neuro-Psychology Laboratory (IRCCS Istituto Auxologico Italiano), Via Pellizza da Volpedo, 41, Milan, Italy; 2 Department of Psychology, Catholic University of Milan, Largo Gemelli, 1, Milan, Italy; 3 Virtual Reality Medical Institute, 30 Clos Chapelle aux Champs, Bte. 1, 3030, Brussels, Belgium

**Keywords:** Psychological Stress, Virtual Reality, Smartphone, Biofeedback

## Abstract

**Background:**

Psychological stress occurs when an individual perceives that environmental demands tax or exceed his or her adaptive capacity. Its association with severe health and emotional diseases, points out the necessity to find new efficient strategies to treat it. Moreover, psychological stress is a very personal problem and requires training focused on the specific needs of individuals. To overcome the above limitations, the INTERSTRESS project suggests the adoption of a new paradigm for e-health - Interreality - that integrates contextualized assessment and treatment within a hybrid environment, bridging the physical and the virtual worlds. According to this premise, the aim of this study is to investigate the advantages of using advanced technologies, in combination with cognitive behavioral therapy (CBT), based on a protocol for reducing psychological stress.

**Methods/Design:**

The study is designed as a randomized controlled trial. It includes three groups of approximately 50 subjects each who suffer from psychological stress: (1) the experimental group, (2) the control group, (3) the waiting list group. Participants included in the experimental group will receive a treatment based on cognitive behavioral techniques combined with virtual reality, biofeedback and mobile phone, while the control group will receive traditional stress management CBT-based training, without the use of new technologies. The wait-list group will be reassessed and compared with the two other groups five weeks after the initial evaluation. After the reassessment, the wait-list patients will randomly receive one of the two other treatments. Psychometric and physiological outcomes will serve as quantitative dependent variables, while subjective reports of participants will be used as the qualitative dependent variable.

**Discussion:**

What we would like to show with the present trial is that bridging virtual experiences, used to learn coping skills and emotional regulation, with real experiences using advanced technologies (virtual reality, advanced sensors and smartphones) is a feasible way to address actual limitations of existing protocols for psychological stress.

**Trial registration:**

http://clinicaltrials.gov/ct2/show/NCT01683617

## Background

According to Cohen and colleagues [1], psychological stress occurs when an individual perceives that environmental demands tax or exceed his or her adaptive capacity. In this view, stressful experiences are conceptualized as person-environment transactions, whose result is dependent on the impact of the external stimulus. This is mediated by the following: (1) the person’s appraisal of the stimulus: when faced with a stimulus, a person evaluates the potential threat (primary appraisal). Primary appraisal is a person’s judgment about the significance of a stimulus as stressful, positive, controllable, challenging or irrelevant; (2) The personal, social and cultural resources available: facing a significant stimulus, a second appraisal follows, which is an assessment of the individual’s coping resources and options. Secondary appraisals address what one can do about the situation; (3) the efficacy of the coping efforts: if required by the appraisal process the individual starts a problem management phase aimed at regulation of the external stimulus.

Extreme levels of stress can have a negative influence on one’s professional life and can disrupt both the social and personal life of an individual. Stress can also cause different physiological and psychological disorders, such as anxiety, chronic headaches, depression, withdrawal symptoms, nausea, phobias, blood pressure problems, heart impairments and others [2-5].

Repeated and early exposure to stress, above all in persons with a particular genetic disposition, as well as high level of trait anxiety, may result in a decreased threshold for developing anxiety [6-8]. Overexcitation and deprivation can influence the affective system and may induce changes in the emotional circuitry of the brain that can contribute to stress-related psychopathology [9]. As underlined by Cohen and colleagues [1], associations between psychological stress and disease have been established, particularly for depression, cardiovascular disease (CVD), and HIV/AIDS. Stressful events influence the pathogenesis of physical diseases by causing continuous negative affective states (potentially leading to feelings of anxiety and depression), which in turn exert direct effects on biological processes, activating the hypothalamic-pituitary-adrenocortical axis (HPA) and the sympathetic adrenal medullary system (SAM) or behavioral patterns that influence disease risks. Exposures to chronic stress are considered the most toxic ones because they are most likely to result in long-term or permanent changes in the emotional, physiological, and behavioral responses that influence susceptibility and the disease course [7,10]. This kind of stress can lead to a loss of productivity and to mental health problems, such as depression and anxiety [11]. Moreover, burnout can result as a long-term consequence of stress [12].

Stress management therapy can help to overcome the counter effects of stress. Usually various techniques are used, including relaxation, interaction, biofeedback and cognitive behavioral therapy (CBT) methods. According to the *Cochrane Database of Systematic Reviews*[13-15], the best validated approach covering both stress management and stress treatment is CBT. Typically, this approach may include individual interventions (10 to 15 sessions) interwoven with didactics. It includes in-session didactic materials and experiential exercises, and out-of-session assignments (practicing relaxation exercises and monitoring stress responses). The intervention focuses on learning to cope well with daily stressors and optimizing one's own use of personal and social resources.

The intervention also encourages emotional expression, replacing doubt appraisals with a sense of confidence by means of cognitive restructuring, and honing skills in anxiety reduction (by progressive muscle relaxation or diaphragmatic breathing and relaxing imagery). The CBT package thus includes both problem-focused (for example, resource optimization and better planning) and emotion-focused (for example, relaxation training, use of emotional support) coping strategies.

Nevertheless, the Cochrane database underlines that “the poor quality of trials, considerable heterogeneity observed between trials and evidence of significant publication bias make the pooled findings insecure.” In other words, even if CBT is the best-validated approach for the treatment of stress [16-19], further clinical research is needed to tune existing protocols and fully exploit its clinical potential.

The trouble with stress is that it is so very personal. Thus, stress-related disorders cannot be explained simply on the basis of the terrible things that happen to people. These disorders depend a great deal on how the person experiencing a stressor is put together, psychologically and physically. So the focus for assessment, prediction and treatment has to be the situational experience of the person. For this aim, the INTERSTRESS project suggests the adoption of a new paradigm for e-health - Interreality, which integrates contextualized assessment and treatment within a hybrid environment, bridging the physical and the virtual worlds [20-24].

The Interreality claim is that bridging virtual experiences (fully controlled by the therapist, and used to learn healthy behaviors and coping skills) with real experiences (the therapist can identify critical situations and assess clinical change), using advanced technologies (virtual worlds, advanced sensors and smartphones) is a feasible way to address the complexity of many mental disorders, including psychological stress. On one hand, patients are continuously assessed in the virtual and real worlds by tracking their behavioral and emotional status in the context of challenging tasks (customization of the therapy according to the characteristics of the patient). On the other hand, feedback is continuously provided to improve patients’ skills through a conditioned association between performance and execution of assigned tasks (improvement of self efficacy). Within the Interreality paradigm, INTERSTRESS aims to create a feasible way to address the current in vivo (real-life) limitations of psychological stress for existing protocols.

In the standard CBT protocol for stress management, imagination and/or exposure evoke emotions, and the meaning of the associated feelings can be changed through reflection and relaxation. The INTERSTRESS project, based on the Interreality paradigm, would suggest an alternative, which is able to control experiences that evoke emotions resulting in meaningful new feelings, which can be reflected upon and eventually changed through reflection and relaxation.

Although CBT focuses directly on modifying dysfunctional thoughts through a rational and deliberate process, INTERSTRESS will focus on modifying them through a more contextualized experiential process. In the INTERSTRESS training, in fact, individuals will be actively involved in the learning process, experiencing stressful situations reproduced in virtual environments and reflecting on the stress level in their daily life with the help of advanced technology, such as smartphones and unobtrusive biosenors.

From the clinical point of view, the INTERSTRESS solution may offer the following innovations to current traditional protocols for stress management: (1) integrated and quantitative assessment of the user’s stress level using biosensors and behavioral analysis: the level of stress will be continuously assessed in the virtual and in the real world by recording the participant's behavioral and emotional status; (2) provision of warnings and motivating feedback to improve self awareness, compliance and long-term outcomes: participants will constantly receive feedback to improve their appraisal and coping skills in an entertaining and motivating fashion.

## Methods/Design

### Aim of the study

The main goal of the INTERSTRESS project is the development of a highly innovative information and computer technology (ICT)-based system for the assessment and management of psychological stress from a strictly context-bound and interactive perspective, which means offering specific tools for addressing the complexity of the phenomenon.

The general aim of the trial is to test the efficacy of the INTERSTRESS service, an advanced ICT-based solution for the diagnosis and treatment of psychological stress. In particular, the purpose is to test the efficacy of the INTERSTRESS service for the objective and quantitative assessment of psychological stress symptoms and to integrate the decision support system (DSS) for treatment planning through data fusion and detection algorithms.

The assessment will be conducted continuously in the virtual and real worlds through the tracking of individuals’ behavioral and emotional status over time in the context of realistic task challenges. On the other hand, the information will be constantly used to improve both the appraisal and the coping skills of the participant through conditioned association between effective performance state and task execution behaviors. The devices are integrated around two subsystems: the clinical platform (indoor treatment, fully controlled by the trainer) and the mobile platform (real-world support, available to the participant and connected to the trainer). Furthermore, from a scientific point of view the INTERSTRESS trial aims to: (1) define a multidimensional approach to the assessment of psychological stress, able to integrate traditional measures with real-time assessment of the quality of the individual experiences, through the integration of biosensors and behavioral analysis; (2) provide support for efficient functioning in everyday life; and (3) verify the efficacy of the proposed methodological approach and of the system for psychological stress prevention, through an evidence-based approach in real-world organizational settings.

### Participants

The present trial is based on a controlled trial, including three groups of approximately 50 individuals who suffer from psychological stress: (1) the experimental group (EG), (2) the control group (CG), and (3) the waiting list (WL) group. Persons who feel stressed at their place of employment and who lack coping skills will be randomly assigned to one of these three groups.

We will recruit up to 150 participants: up to 75 teachers will be collected by *Istituto Auxologico Italiano* (AUXO) in public school institutes in Milan, and up to 75 nurses will be recruited by the National Council of Research (CNR) in a public hospital in Messina, Italy. The number of participants (sample size) was calculated in order to have an effect size sufficient to prove the effect of the training, reducing type II (false negative) statistical errors. Regarding type I errors (false positive), a test threshold of 0.05 will be used.

In order to be included in the study, individuals have to meet the following criteria: (1) no Axis-I disorders as defined in the *Diagnostic and Statistical Manual of Mental Disorders*, *fourth edition* (DSM-IV-TR); (2) age between 25 and 60 years; (3) no psychotherapy received for their psychological stress; (4) no pharmacotherapy; (5) no history of neurological diseases, psychosis, alcohol or drug dependence; and (6) no migraine, headache, or vestibular abnormalities. Both male and female patients will be included. Before participating in the study, each participant will be provided with written information about the study and will be required to give written consent for inclusion in the study.

### Questionnaires

For the assessment of the stress management training outcome, we will administer questionnaires at different time points and for different categories of interest.

#### Clinical assessment

In order to exclude participants suffering from DSM-IV-TR Axis-I disorders, individuals will be assessed before the start of the training by a Masters-level charted psychologist or a PhD-level psychotherapist using a semi-structured interview, the mini-international neuropsychiatric interview (MINI) [25,26]. The MINI is a short diagnostic structured interview, which enables researchers to make diagnoses of psychiatric disorders according to DSM-IV or International Classification of Diseases (ICD)-10. The administration time of the interview is approximately 15 minutes. The interview was designed for epidemiological studies and multicenter clinical trials.

#### Technological skills assessment

At the beginning of the training a self-assessment scale will be used to evaluate patients’ technological abilities. The four-item questionnaire was created to assess individuals’ perceived technological skills in the use of personal computers and smartphones.

#### Psychometric assessment

The following questionnaires will be administered to each participant at pre-treatment, upon completion of the trial, and after an 18-month follow-up period. The psychological stress measure (PSM) [27,28] consists of 49 items, based on the various individual perceptions of cognitive, physiological, and behavioral state of subjects. The PSM provides a global score of stress and some partial sub-scores. The individual is asked to answer on the basis of how he or she has been feeling in the last 4 to 5 days. The global score of the PSM is compared with ground truth scores, which gives a threshold cutoff on the basis of gender (103 for male and 110 for female subjects).

The perceived stress scale (PSS) [29] is a 10-item self-reported measure designed to deal with the degree to which situations in an individual’s life are appraised as stressful. It was originally developed as a 14-item scale that assessed the perception of stressful experiences over the previous month using a 5-point Likert scale. Later, the authors reported that the 10-item version (PSS-10) showed stronger psychometric characteristics in comparison to the 14-item scale (Cohen and Williamson, 1988).

The questionnaire, coping orientation to problems experienced inventory (COPE) [30,31], was developed to assess a broad range of coping responses. It presents 14 scales all assessing different coping dimensions such as, active coping, planning, and using instrumental support. Each scale contains two items, for a total of sixty items altogether. This inventory can be used to assess trait coping (the usual way people cope with stress in everyday life) and state coping (the particular way people cope with a specific stressful situation).

The Pittsburgh sleep quality index (PSQI) [32,33] assesses the sleep quality and disturbance over a one-month time interval. Nineteen items generate seven scales: subjective sleep quality, sleep latency, sleep duration, habitual sleep efficiency, sleep disturbance, use of sleeping medication and daytime dysfunction. It also results in a global score.

The state-trait anxiety inventory (STAI) [34,35] consists of two scales containing twenty items each that measure anxiety in adults. The STAI clearly differentiates between the temporary condition of ‘state anxiety’ (STAI form Y-1, also known as STAI-Y1) and the more general and long-standing quality of ‘trait anxiety’ (STAI form Y-2*,* also known as STAI-Y2). For the initial and the final assessment in the trial we will use the STAI Y-2, that is, the trait version of the STAI, which measures characteristic tendencies towards anxiety.

The satisfaction with life scale (SWLS) [36,37] is a measure of life satisfaction (subjective well-being). The 5-item questionnaire is designed to measure global cognitive judgments of satisfaction with one`s own life.

Individuals will also be assessed at the beginning and at the end of each of the eight training sessions using the following questionnaires. The STAI Y-1 [34,35] addresses the individual’s state of anxiety, which could be defined as a temporary emotional condition characterized by apprehension, tension, and fear about a particular situation or activity. This inventory consists a of a 20-item scale, similar to the STAI Y-2. The visual analog scale for anxiety (VAS-A) is an instrument that measures anxiety across a continuum. It is a horizontal line, 100 mm in length, anchored by word descriptors at each end (no anxiety; very severe anxiety). Individuals mark on the line the point that they feel represents their perception of their own current state. The VAS-A score is determined by measuring in millimeters from the left-hand end of the line to the point that the person marks.

### Psychophysiological assessment

Clinical and mobile settings, planned in the INTERSTRESS training, require different participants’ psychophysiological assessment as shown specifically below.

#### Clinical setting

Psychophysiological data (heart and respiratory rates) will be obtained from a cardiovascular belt developed for this project by Pisa National Centre of research. These belts will be connected to a multi-modality device for real-time computerized data acquisition through a Bluetooth connection. Heart and respiratory channels will be acquired at a 100-Hz sampling rate. Cardiorespiratory activity will be monitored to evaluate voluntary and autonomic effects of respiration on heart rate (HR) in physical and virtual environmental interactions, analyzing the R-R interval (from an electrocardiogram (ECG)) and the breath respiration (BR) (from a chest-strip sensor) and their interaction. Furthermore, spectral methods for assessment of standard indexes of heart rate variability (HRV) will be used to evaluate the response of the autonomic nervous system. Spectral analysis will be performed by means of autoregressive (AR) spectral methods with custom software. The Levinson-Durbin recursion will be used to identify the coefficients of an autoregressive model. The AR spectral decomposition procedure, for example, will be applied to calculate the power of the oscillations embedded in the series. Moreover, the quantification of respiratory sinus arrhythmia (RSA) will provide information about the mechanisms involved in respiratory coupling.

#### Mobile setting

During the training, participants in the EG will also be monitored outside of the trainer’s office through a cardiovascular belt developed by University of Pisa that will send their psychophysiological data to a web-based Central Database Repository though a smartphone. Electrocardiography will be used to monitor heart rate and cardiac cycle through the inter-beat-interval (IBI). These cardiovascular data will be used to monitor individual psychophysiological changes related to psychological stress in real-life settings. Moreover, HR signals will be integrated into an application on the participants’ smartphones, to give them the possibility to perform biofeedback exercises, base on HRV, every time they are required [38].

### Data collection

Epidemiological, clinical and psychophysiological data from each individual will be recorded by trainers across the study using advanced technologies. Data collected in the clinical and in the mobile setting will be stored in the web-based Central Database Repository. Security and privacy issues will be taken care of according to the respective situation and the consent of the participant. At the end of the training, the trainers will arrange a review appointment with the participant, both in the EG and the CG. Clinical follow up data (scores from the same psychological questionnaire given at the end of the training) will be collected at those time points.

### Experimental design

In order to study the efficacy of the INTERSTRESS training, a between-subject design will be used with two experimental conditions and repeated measurements (pre-training and post-training). Specifically, we will compare the following conditions: (1) individuals in the EG will receive training based on CBT and new technologies (virtual reality (VR), VR combined with biofeedback, and mobile phones). Relaxation will be induced through the immersion in different virtual environments (for example, a lake), which will be customized with different pre-recorded audio narratives that describe the specific setting and that guide the execution of a series of relaxation exercises. During biofeedback exercises a wearable biosensor system will provide suggestions to the trainer based on the reactions of the participants, and the biosensor data will directly modify the VR experience in real time; (2) individuals in the CG will receive training based on traditional CBT for stress management, without the use of new technologies. Relaxation will be induced by guided imagery, through auditory narratives; (3) individuals in the WL group will be reassessed and compared with the two other groups five weeks after the initial evaluation. After the reassessment, the WL patients will randomly receive one of the two other treatments.

**Figure 1:**
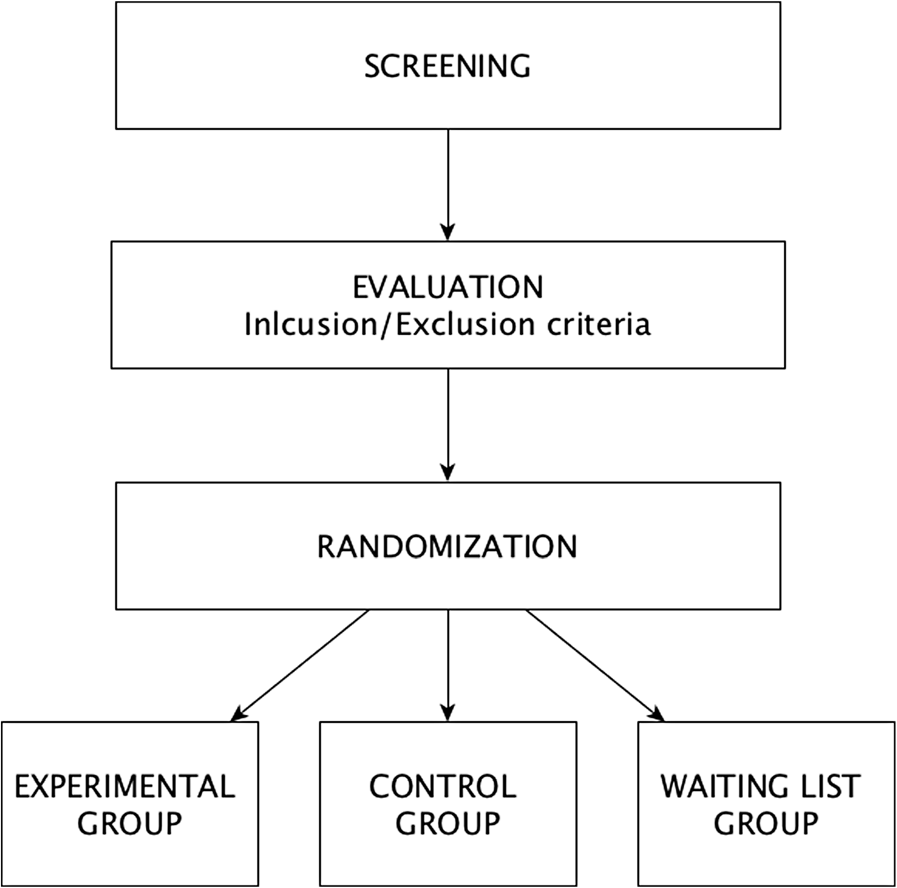
CONSORT Flow diagram.

Psychometric and psychophysiological outcomes will serve as quantitative dependent variables, and subjective reports of participants will be used as qualitative dependent variables (Figure 1).

### Randomization

The present trial is a randomized controlled trial. Participants will be randomly assigned to one of the three groups of the trial.

### Hardware

Different hardware elements will be used in each setting of the INTESTRESS training, as shown specifically below.

#### Clinical setting

**Figure 2:**
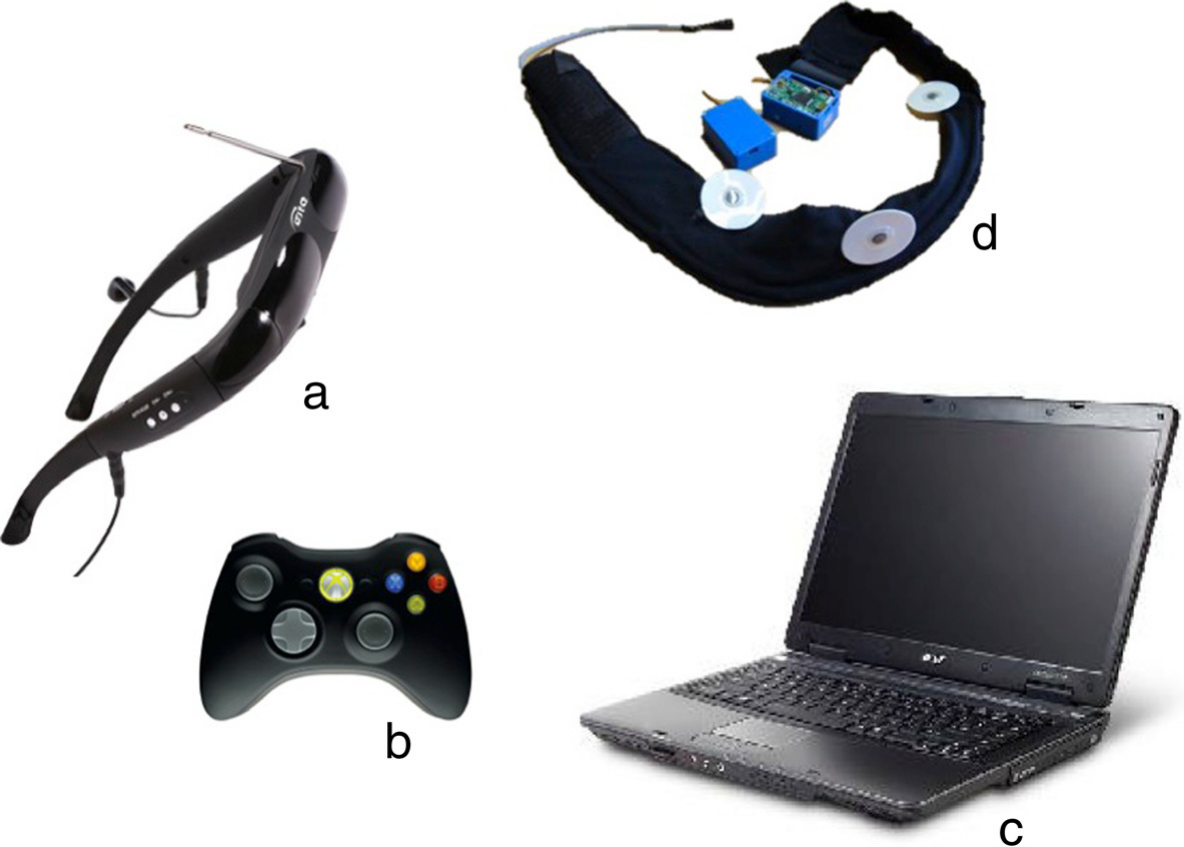
**Clinical setting hardware units. a**) the head-mounted display (Vuzix VR Bundle with twin high-resolution 640 × 480 LCD displays, iWear^®^ 3D-compliant); **b**) the joypad (Xbox controller); **c**) the portable computer (ACER ASPIRE with Intel^®^ Core™i5, graphic processor Nvidia GeForce GT 540M and Bluetooth support); **d**) the cardiovascular belt (Pisa National Centre of Research).

A cardiovascular belt, developed by Pisa National Centre of Research will be used to measure HR and respiration activity. It will consist of independent lycra-based wearable bands for the examination of the physiological (HR, HRV and BR) and behavioral signals (activity classification through motion analysis). We will use a VR control unit, the ACER ASPIRE portable computer with CPU Intel^®^ Core™i5, graphic processor Nvidia GeForce GT 540M and Bluetooth support; a head-mounted display: Vuzix VR Bundle with twin high-resolution 640 × 480 (920,000 pixels) LCD displays, iWear^®^ 3D-compliant; and a joypad (Xbox controller) (Figure 2).

#### Mobile setting

**Figure 3:**
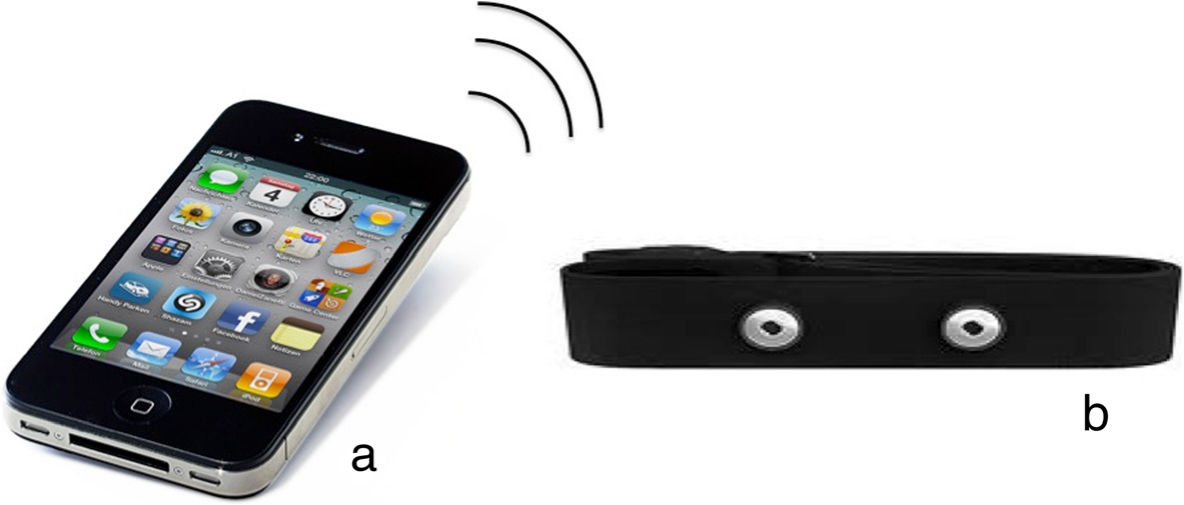
**Mobile setting hardware units. a**) the smartphone (iPhone 4S); **b**) the shimmer, (University of Pisa).

A shimmer, which is a small wireless sensor platform developed by University of Pisa, will be used to record and transmit psychophysiological (ECG) and kinematic data in real time. We will use the iPhone 4S smartphone (Figure 3).

#### Home setting

We will use a portable computer (ACER ASPIRE 5742G-484G64MNKK) with Internet connection. Only participants in the EG will receive technological devices for use in the mobile and home settings, whereas those in the CG will instead receive a paper-and-pencil diary and a compact disc (CD) audio with relaxing narratives. Trainers and participants in the EG and the CG will have the possibility to access the Patient Management System (PMS). This web platform (content management system) will play the role of the front-end component used by the trainer and the participant to interact with the whole INTERTRESS system. Through PMS, both the trainer and the participant can access the relevant data stored in the central database, helping to monitor the evolution of the stress levels in patients under treatment, and to organize the therapist-patient interaction (scheduling of appointments, homework assigned to the patient, et cetera).

### Virtual environments

#### Virtual scenarios for stress exposure

VR can be considered one of the preferred environments for the empowerment processes, since it is a special, sheltered setting where users can start to explore and act without feeling threatened. In this sense, the virtual experience is an empowering environment that psychologists provide for their clients. In addition, VR allows the situation to be graded so the user can start at the easiest level and progress to the most difficult. Gradually, because of the knowledge and control afforded by interaction in the virtual world, the participants will be able to face the real world.

Starting from a literature review focused on exploring the daily life situations that produce stress [39-41], and specific focus groups, a list of work-related stressful situations has been gathered. According to the literature review and to the results of a qualitative analysis, the following seven virtual stressful scenarios were created for the protocol, focused on the first class of our sample (teachers): (1) workload; (2) class management; (3) coping with parent’s criticism; (4) relationship with boss; (5) coping with parent’s handling efforts; (6) relationship with coworkers; and (7) conflict management.

**Figure 4:**
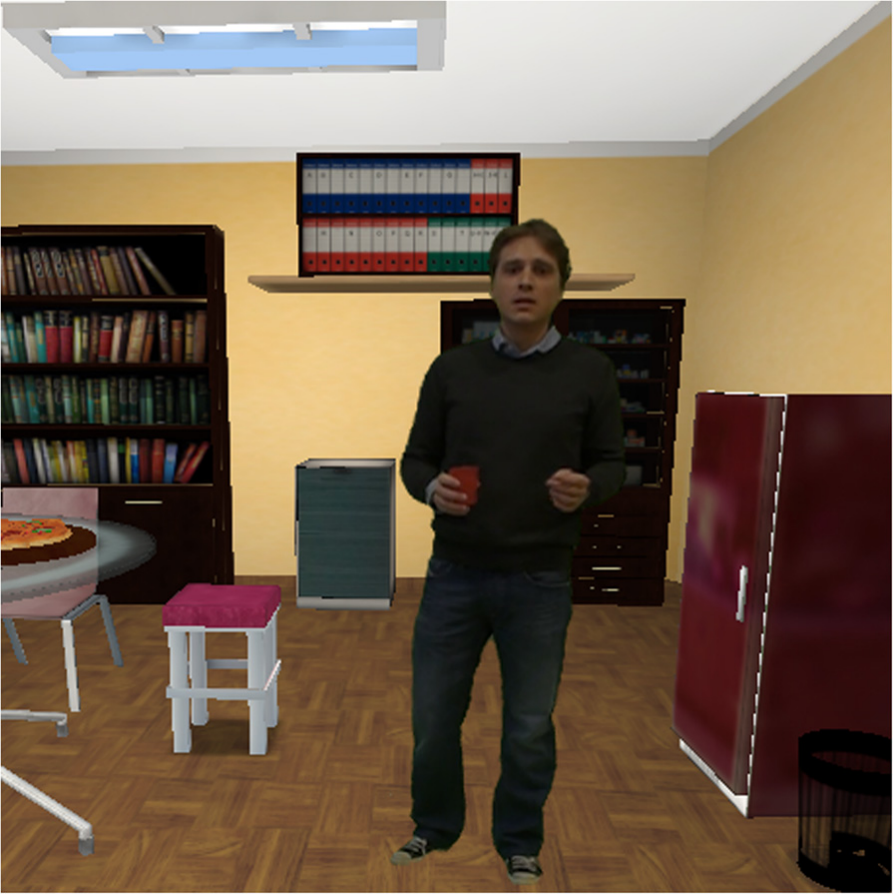
Virtual reality stress scenario screenshot.

Thus, the storyboards for each stressful scenario were written and the virtual environments were created according to these storyboards. The stressful scenarios were then played by real actors, who were inserted into the virtual environments after a video post-production (Figure 4). For the EG the stressful scenarios will be played by NeuroVR 2, a cost-free VR platform [42,43]. The efficacy of these selected stressful scenarios in eliciting negative emotional responses was first tested on a sample of ten teachers.

Stressful virtual scenarios for the other sample of the trial (nurses) are currently being created following the same procedure. The virtual environments created will include stressful situations that could be experienced by nurses at work, such as to be reproached by colleagues, to manage an emergency and to cope with patients’ criticism.

As already explained, the CG will not be exposed to VR. They will receive the same script of the seven stressful scenarios through an audio version (guided imagery). Participants will be asked to close their eyes and to imagine themselves, as vividly as possible, during the situations described, following an audio narrative matching the VR experiences.

#### Relaxing virtual environments

In the last years, VR has been used in different clinical protocols to facilitate relaxation processes in stressed or anxious subjects [44-46] by visually presenting key relaxing images. The advantage of VR compared to other tools, such as CDs or digital video discs (DVDs), which are traditionally used in CBT, is the ability to induce a sense of presence in the users, which can be defined as the ‘feeling of being in a world that exists outside of the self ’ [47], increasing the efficacy of the experience. The visual presentation of a virtual calm scenario can facilitate subjects' practice and mastery of relaxation, making the experience more vivid and real than the one that most subjects can create using their own imagination and memory, and triggering a broad empowerment process within the experience induced by a high sense of presence [48,49].

**Figure 5:**
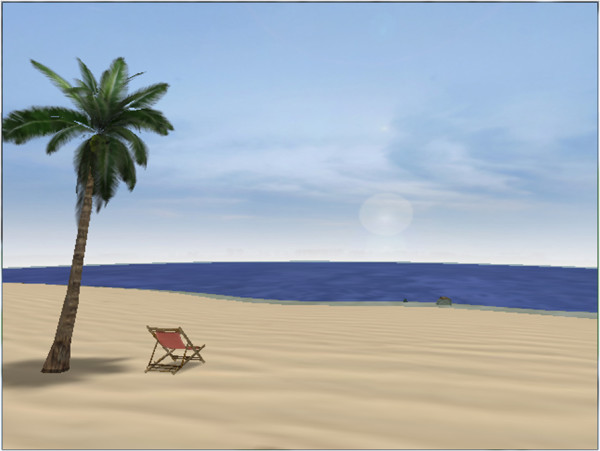
Virtual reality relaxing scenario screenshot.

In the INTERSTRESS trial, for the relaxation training, relaxing environments will be presented together with relaxing audio narratives based on guided imagery procedures and developed according to emotive engineering principles [50,51]. The INTERSTRESS relaxation environments were created on the basis of similar virtual relaxing environments that were used and validated in previous studies [45,52-54] and they include, for example, a beach, a lake, a campfire, a mountain summit, and a desert (Figure 5). In the clinical setting they can be viewed through head-mounted displays, whereas in the mobile setting they can be watched on a smartphone display (audio or video with audio). The CG will receive the same relaxing exercises in an audio version through an audio CD.

### Use of smartphones

Through the smartphones participants will have the opportunity to receive warning and feedback about their stress level and to perform relaxing or biofeedback exercises. This technology offers the chance to close the gap between in-office and out-of-office (or homework) sessions, improving compliance and long-term outcomes. Recent work [53,55-57] verified the efficacy of a stress management protocol supported by the use of mobile phones. According to that study, the advantages of using a mobile approach to reduce stress could be an incremental acquisition of coping skills in an autonomous way, a ubiquitous and effective support in facing daily stressful situations, an enhancement of people’s compliance and the possibility of living graded-exposure experiences while overcoming the difficulties related to the application of coping techniques within a clinical setting. Moreover, thanks to the recent progress in the sophistication and in the usability of biosensor technology wirelessly connected to mobile platform, it is now possible to set up a multimodal assessment of stress levels, including psychological, physiological, behavioral, and contextual data [58-61].

### Use of biofeedback combined with virtual reality

Biofeedback has demonstrated value for stress-reduction training [62,63]. Unfortunately, one of the main limitations of traditional biofeedback is that subjects receive very simple audio and video feedback information from a computer that processes their physiological data, such as blood pressure, HR, skin temperature, sweat gland activity, and muscle tension, in real time. As demonstrated in a previous study [53], an immersive virtual relaxing environment helps users to master the complex training of relaxation, reinforced by using physiological data to modify specific features of the virtual environment in real time.

### Protocol

The protocol will be based on 5 weeks of stress management training (two sessions per week). During each week participants in the EG and the CG will meet a psychologist in two sessions. Our protocol is based on CBT in general, and specifically on the stress management program of Kaluza [64] and on the stress inoculation training of Meichenbaum [65]. As before, different settings are planned for the training; these are shown below.

#### Clinical setting

During the session, the participant will be in a quiet room, sitting in a comfortable chair. Every session will last about 50 minutes and will consist of a first part with an introduction and a discussion about homework, a central part with exposition to a stressful scenario, followed by cognitive restructuring aimed at teaching participants about useful coping strategies related to the specific stressful situation. After this exposition and discussion, there will be a final part for relaxation training, and finally a debriefing about the session.

#### Home setting

**Figure 6:**
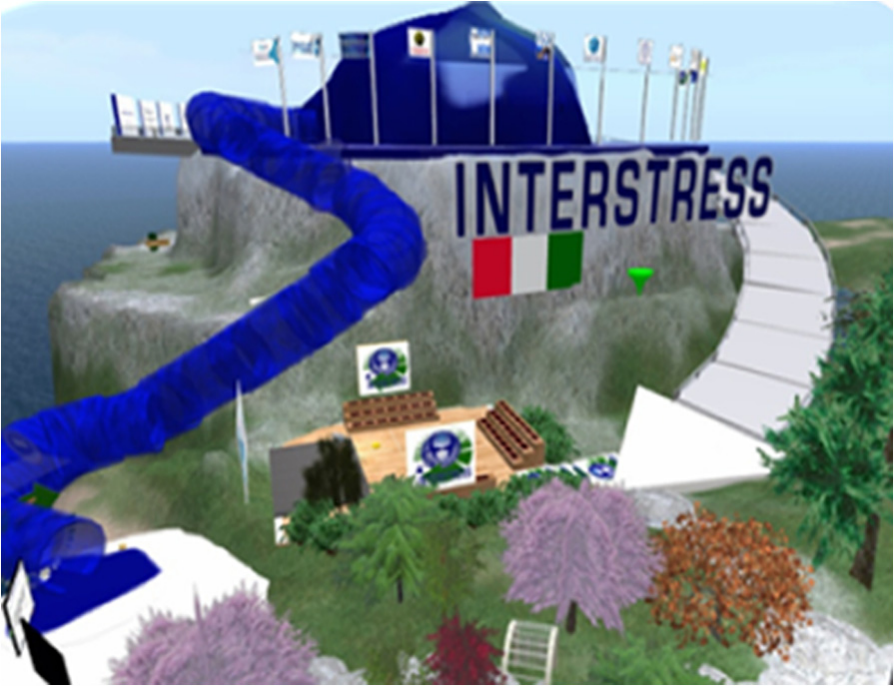
Learning island screenshot.

Participants will have the possibility to access the contents useful for stress management logging in from their personal computer (PC) via Second Life and visiting the INTERSTRESS Learning Island [61]. Simply by exploring virtual worlds, they can learn different aspects related to psychological stress, such as its main causes, its symptoms, stress management and coping strategies (Figure 6).

#### Mobile setting

Participants will be able to rehearse/relax outside the psychologist’s office using a smartphone application developed for this specific purpose. To improve the efficacy of the experience with the clinician, during the homework the mobile system will present a non-navigable version of the same VR environment experienced during the training in the clinic setting. Through the smartphone, participants will also be taught how to become aware of their physiological levels of stress. On the mobile phone they will have the possibility to observe a simple graphic of their stress level on that day and during their week. Moreover, the mobile application provides warning signals when critical thresholds are overcome, giving the participant the opportunity to perform a relaxing exercise to reduce his or her stress level.

A detailed description of the protocol for the EG and the CG follows: In the EG (see Table 1), participants will receive training based on cognitive behavioral techniques combined with VR, biofeedback and use of mobile phones.

**Table 1:** Study protocol for the experimental group

**Intake session**	**Introduction of the training**		**Clinical interview (MINI)**	**Give STAI Y2 and SWLS**		**Questionnaire about participant’s expectations and motivations**	**Exposition to a neutral virtual environment**	**Explanation of how to use the PDA, Second Life and the PMS**
**Session I**	Introduction	Physiological assessment	Give STAI Y1 and VAS-A (baseline)	Exposition to stressful virtual environment n1	Give STAI Y1 and VAS-A (after each scenario)	Repeated for all the 7 stressful environments + neutral one + cognitive task	Exposition to a relaxing virtual environment	Final debriefing
Assessment session								
**Session II**	**Preliminary phase**		**Pre-training**	**Training**			**Post-training**	**Final phase**
Training session	Introduction	Give STAI Y1 and VAS-A	Physiological assessment	Exposition to stressful virtual environment	Cognitive restructuring	Exposition to a relaxing virtual environment	Give STAI Y1 and VAS-A	Delivery of homework and debriefing
**Session III**	**Preliminary phase**		**Pre-training**	**Training**			**Post-training**	**Final phase**
Training session	Introduction	Give STAI Y1 and VAS-A	Physiological assessment	Exposition to stressful virtual environment	Cognitive restructuring	Biofeedback in virtual reality	Give STAI Y1 and VAS-A	Delivery of homework and debriefing
**Session IV**	**Preliminary phase**		**Pre-training**	**Training**			**Post-training**	**Final phase**
Training session	Introduction	Give STAI Y1 and VAS-A	Physiological assessment	Exposition to stressful virtual environment	Cognitive restructuring	Exposition to a relaxing virtual environment	Give STAI Y1 and VAS-A	Delivery of homework and debriefing
**Session V**	**Preliminary phase**		**Pre-training**	**Training**			**Post-training**	**Final phase**
Training session	Introduction	Give STAI Y1 and VAS-A	Physiological assessment	Exposition to stressful virtual environment	Cognitive restructuring	Biofeedback in virtual reality	Give STAI Y1 and VAS-A	Delivery of homework and debriefing
**Session VI**	**Preliminary phase**		**Pre-training**	**Training**			**Post-training**	**Final phase**
Training session	Introduction	Give STAI Y1 and VAS-A	Physiological assessment	Exposition to stressful virtual environment	Cognitive restructuring	Exposition to a relaxing virtual environment	Give STAI Y1 and VAS-A	Delivery of homework and debriefing
**Session VII**	**Preliminary phase**		**Pre-training**	**Training**			**Post-training**	**Final phase**
Training session	Introduction	Give STAI Y1 and VAS-A	Physiological assessment	Exposition to stressful virtual environment	Cognitive restructuring	Biofeedback in virtual reality	Give STAI Y1 and VAS-A	Delivery of homework and debriefing
**Session VIII**	**Preliminary phase**		**Pre-training**	**Training**			**Post-training**	**Final phase**
Training session	Introduction	Give STAI Y1 and VAS-A	Physiological assessment	Exposition to stressful virtual environment	Cognitive restructuring	Exposition to a relaxing virtual environment	Give STAI Y1 and VAS-A	Delivery of homework and debriefing
**Session IX**	**Preliminary phase**		**Pre-training**	**Training**			**Post-training**	**Final phase**
Training session	Introduction	Give STAI Y1 and VAS-A	Physiological assessment	Exposition to stressful virtual environment	Cognitive restructuring	Biofeedback in virtual reality	Give STAI Y1 and VAS-A	Delivery of homework and debriefing
**Session X**	Follow up of questionnaires used in the evaluation phase	Physiological assessment	Give STAI Y1 and VAS-A	Exposition to stressful virtual environment n1	Give STAI Y1 and VAS-A	Repeated for all the 7 stressful environments + neutral one + cognitive task	Exposition to a relaxing Virtual Environment	Final debriefing
Final session								

*MINI*, mini-international neuropsychiatric interview; *STAI*, state-trait anxiety inventory; *SWLS*, satisfaction with life scale; *PDA*, personal digital assistant; *PMS*, patient management system; *VAS-A*, visual analog scale for anxiety.

The protocol has the following schema.

Intake session: before this session, in order to assess the participants’ stress features, they are asked to fill out self-completion questionnaires at home (MSP, COPE, PSS, PSQI, STAI, SWLS and a questionnaire about technological abilities) and give these to the clinician at the beginning of the session. During the intake session, the participant will meet the trainer, who will explain in detail the training purpose and procedures, and will finalize the assessment. After discussion and explanation of the aims and methods of the training, each participant will be provided with written information about the study and will be asked to provide written consent for inclusion. Then, the participant will be assessed through clinical interview (MINI) in order to verify if he or she could be included in the training.

After this preliminary phase, if the participant satisfies the inclusion criteria, the psychologist will introduce the specific processes of the training, which will be structured into ten sessions (two per week, each one lasting one hour) where technologies will be employed as training tools. Each session will be divided into four parts: homework checking and dialogue with the participant on difficulties experienced in the week between the previous session and the current session; exploration of virtual environments to practice coping skills during stressful situations; comments about the experience (debriefing and discussion of positive use of coping skills, noted areas of difficulty and subjective perception of stress versus objective measurement of stress by the means of biosensors); and relaxation training and new homework assignments. Then, for familiarization with VR, the clinician will expose the participant to a neutral virtual environment. After this exposition, the trainer will explain that biosensors will be worn during the training to monitor physiological parameters, to track the emotional/heath status, and to directly influence experience in the virtual world. The session will be concluded with a detailed explanation of how to use the portable biosensors and the smartphone during the following assessment week. The clinician will also explain how to use the patient management system through the computer at home and the three-dimensional virtual spaces (Learning Island) in Second Life during all the training.

Assessment session (session 1): this will start with a discussion with the clinician about the assessment week. Then, after a brief introduction to the specific content of this session, the physiological baseline of the participant will be recorded for 3 minutes. In order to measure the psychological variations occurring during the different stressful environmental exposures, subjects will complete the VAS-A and STAI-Y1 at baseline and after each scenario. During the stressful exposition, the participant’s physiological measurements will also be recorded. In addition to the seven stressful scenarios, which replicate daily life stressors, each participant will be assessed in a neutral virtual environment and in one in which he or she will complete a cognitive task in front of a virtual audience. These situations will be used to assess the maximum and minimum stressor. After the virtual assessment, the participant will be exposed to a relaxing virtual environment. At the end of this session, the clinician will explain to the participant the homework to be completed using the smartphone and the PC .

Training session (sessions 2 to 9): these sessions will be dedicated to teaching participants to cope with stress through cognitive restructuring techniques and the teaching of relaxation exercises. Each session will last about one hour and be divided into four parts: homework checking; exposition to a stressful scenario and cognitive restructuring with the clinician about the experience; relaxation; and homework assignment. The clinician will decide with the participant, which specific stress scenarios he or she will focus on during the training. The participant will be virtually exposed to the selected stress, working on it with the clinicians during two consecutive sessions. Relaxation will be induced through immersion in different virtual environments (for example, a lake), which will be customized with different pre-recorded audio narratives that describe the specific setting and guide the execution of a series of relaxation exercises. Relaxation will be induced by navigation in a virtual environment in which the user may move and interact, following auditory narratives that support images. Participants will be immersed in different environments (lake, beach, desert, et cetera), each one associated with different pre-recorded audio narratives.

During sessions, the EG will be alternately exposed to relaxing VR environments and to VR biofeedback. The psychologist will provide explanations about biofeedback, the role of biofeedback methodology and the participant's role and involvement. After an introductory explanation, the trainer will start the biofeedback session, while explaining how the physiological signals change with variations in physiologic activity, and how these may be associated with slight differences in subjective feeling states. The participant will be instructed about relaxation and various strategies used to achieve these states during exercises in virtual reality environments that will last about 10 minutes. At the end of the session, the psychologist will discuss the participant's response to the biofeedback experience and will encourage his participation and interaction in the experience. Traditional relaxation exercises will be alternated during sessions with biofeedback.

Follow up session (session 10): in order to verify the efficacy of the training, during the final session, participants will be reassessed through the administration of questionnaires to measure their stress and anxiety level (MSP, PSS, COPE, and STAI-Y2) and their perceived quality of life (PSQI and SWLS). Moreover, participants will again be exposed to the stressful scenarios, following the same procedure as in the assessment session (session 1). At the end of this assessment, the clinician will discuss with the participant the training and its efficacy, and eventually the difficulties he or she has experienced.

In the CG (see Table 2), participants will receive training based on traditional cognitive behavioral techniques. The protocol will follow the same structure as that used in the EG, but without the use of new technologies and biofeedback.

**Table 2:** Study protocol for the control group

**Intake session**	**Introduction of the training**		**Clinical interview (MINI)**	**Give STAI Y2 and SWLS**		**Questionnaire about participant’s expectations and motivations**	**Explanation of how to use the diary**	
**Session I**	Introduction	Physiological assessment	Give STAI Y1 and VAS-A (baseline)	Guided imagery to stressful environment n1	Give STAI Y1 and VAS-A (after each scenario)	Repeated for all the 7 stressful environments + neutral one + cognitive task	Exposition to a relaxing virtual environment	Final debriefing
Assessment session								
**Session II**	**Preliminary phase**		**Pre-training**	**Training**			**Post-training**	**Final phase**
Training session	Introduction	Give STAI Y1 and VAS-A	Physiological assessment	Guided imagery to stressful environment	Cognitive restructuring	Guided imagery to a relaxing Environment	Give STAI Y1 and VAS-A	Delivery of homework and debriefing
**Session III**	**Preliminary phase**		**Pre-training**	**Training**			**Post-training**	**Final phase**
Training session	Introduction	Give STAI Y1 and VAS-A	Physiological assessment	Guided imagery to stressful environment	Cognitive restructuring	Guided imagery to a relaxing environment	Give STAI Y1 and VAS-A	Delivery of homework and debriefing
**Session IV**	**Preliminary phase**		**Pre-training**	**Training**			**Post-training**	**Final phase**
Training session	Introduction	Give STAI Y1 and VAS-A	Physiological assessment	Guided imagery to stressful environment	Cognitive restructuring	Guided imagery to a relaxing environment	Give STAI Y1 and VAS-A	Delivery of homework and debriefing
**Session V**	**Preliminary phase**		**Pre-training**	**Training**			**Post-training**	**Final phase**
Training session	Introduction	Give STAI Y1 and VAS-A	Physiological assessment	Guided imagery to stressful environment	Cognitive restructuring	guided imagery to a relaxing environment	Give STAI Y1 and VAS-A	Delivery of homework and debriefing
**Session VI**	**Preliminary phase**		**Pre-training**	**Training**			**Post-training**	**Final phase**
Training session	Introduction	Give STAI Y1 and VAS-A	Physiological assessment	Guided imagery to stressful environment	Cognitive restructuring	guided imagery to a relaxing environment	Give STAI Y1 and VAS-A	Delivery of homework and debriefing
**Session VII**	**Preliminary phase**		**Pre-training**	**Training**			**Post-training**	**Final phase**
Training session	Introduction	Give STAI Y1 and VAS-A	Physiological assessment	Guided imagery to stressful environment	Cognitive restructuring	Guided imagery to a relaxing environment	Give STAI Y1 and VAS-A	Delivery of homework and debriefing
**Session VIII**	**Preliminary phase**		**Pre-training**	**Training**			**Post-training**	**Final phase**
Training session	Introduction	Give STAI Y1 and VAS-A	Physiological assessment	Guided imagery to stressful environment	Cognitive restructuring	Guided imagery to a relaxing environment	Give STAI Y1 and VAS-A	Delivery of homework and debriefing
**Session IX**	**Preliminary phase**		**Pre-training**	**Training**			**Post-training**	**Final phase**
Training session	Introduction	Give STAI Y1 and VAS-A	Physiological assessment	Guided imagery to stressful environment	Cognitive restructuring	Guided imagery to a relaxing environment	Give STAI Y1 and VAS-A	Delivery of homework and debriefing
**Session X**	Follow up of questionnaires used in the evaluation phase	Physiological assessment	Give STAI Y1 and VAS-A	Guided imagery to stressful environment n 1	Give STAI Y1 and VAS-A	Repeated for all the 7 stressful environments + neutral one + cognitive task	Exposition to a relaxing virtual environment	Final debriefing
Final session								

*MINI*, mini-international neuropsychiatric interview; *STAI*, state-trait anxiety inventory; *SWLS*, satisfaction with life scale; *VAS-A*, visual analog scale for anxiety.

Guided imagery will be employed for stress exposition in the CG and will also be used for teaching relaxation exercises, instead of the virtual environments. The trainer will play an audio tape with the chosen relaxing place or stressful situation, while the participant sits comfortably in a chair with eyes closed. Different stimulus propositions are included in the audio (auditory, visual, tactile, cutaneous, olfactory, and vestibular) and everything is described in detail, so that a realistic and vivid exposure may be possible.

Participants included in the CG will not use the smartphone for stress assessment, but will use a traditional diary, in which they will mark every stressful event during their assessment week. Moreover, instead of Second Life, participants will receive a book about stress, with the same contents included in those virtual environments. In order to obtain comparable measurements with the EG, participants in the CG will be assessed during the training with the same questionnaires and their physiological measurements will be recorded during each session.

### Follow up assessment

An 18-month follow up is planned in order to verify the efficacy of the training over a long-term period.

### Trial analysis

Data analyses will be carried out using SPSS software. Descriptive methods will be used to demonstrate the consistency of the two groups, to describe participants' characteristics and to report levels of participation and drop out. Analysis of variance will be used to evaluate baseline characteristics of the two groups involved in the study, overall significance of improvement across outcome measures, and drop outs versus maintainers. For each participant, change in psychometric and physiological measures will be calculated and analyzed using the *t*-test for matched groups.

### Ethics approval

This study was approved by Ethics Committee in the *Istituto Auxologico Italiano* of Milan.

## Discussion

Despite CBT being the best validated approach for the treatment of stress [16-19], further clinical research is needed to refine existing protocols and fully exploit its clinical potential. Stress-related disorders cannot be explained simply on the basis of the terrible things that happen to people. They depend a great deal on how the person experiencing a stressor is put together, psychologically and physically. So the focus for assessment, prediction and treatment has to be the situational experience of the user. To overcome these limitations, the INTERSTRESS project suggests the adoption of a new paradigm for e-health, interreality, which integrates contextualized assessment and treatment within a hybrid environment, bridging the physical and virtual world [20-23].

What we would like to show with the present trial is that to bridge the use of virtual experiences, which are fully controlled by the trainer, with real experiences, could be effective for teaching coping skills and emotional regulation. This will allow for both the identification of any critical stressors and assessment of what has been learned by using advanced technologies (VR, advanced sensors and smartphones). The aim of this proposal is to create a feasible way to address the actual limitations of the existing protocols for the treatment of psychological stress.

We believe that the use of this innovative paradigm represents a promising approach, because it enhances the quality of assessment and training of psychological stress within a hybrid, closed-loop empowering experience bridging physical and virtual worlds. Our outcome measures are well-suited to measuring both the subjective and objective impact related to the intervention and the presence of the CG, which will adopt a traditional CBT protocol for psychological stress, to guarantee that the final results are truly attributable to the intervention. This controlled trial will be able to evaluate the effects of the use of the INTERSTRESS solution in the assessment and training of psychological stress, while preserving the benefits of randomization to reduce bias. Its design takes into account the need for internal and external validity and that the results are attributable to the intervention.

### Trial status

Patient recruitment was ongoing at the time of manuscript submission. Data collection will continue at least until November 2013.

## Abbreviations

AR: Autoregressive; AUXO: *Istituto Auxologico Italiano*; BR: Breathing rate; CBT: Cognitive behavioral therapy; CD: Compact disc; CG: Control group; CNR: National Council of Research; COPE: Coping orientation to problems experienced inventory; CVD: Cardiovascular disease; DSM-IV-TR: *Diagnostic and Statistical Manual of Mental Disorders, fourth edition*; DSS: Decision support system; DVD: Digital video disc; ECG: Electrocardiogram; EG: Experimental group; HPA: Hypothalamic-pituitary-adrenocortical axis (HPA); HR: Heart rate; HRV: Heart rate variability; IBI: Inter-beat interval; ICD: International Classification of Diseases; ICT: Information and computer technology; MINI: Mini-international neuropsychiatric interview; PBS: Personal biomonitoring system; PC: Personal computer; PDA: Personal digital assistants; PMS: Patient management system; PSM: Psychological stress measure; PSQI: Pittsburgh sleep quality index; PSS: Perceived stress scale; RSA: Respiratory sinus arrhythmia; SAM: Sympathetic adrenal medullary system; STAI: State-trait anxiety inventory; SWLS: Satisfaction with life scale; VAS-A: Visual analog scale for anxiety; VR: Virtual reality; WL: Waiting list.

## Competing interest

The authors declare that they have no competing interests.

## Authors’ contribution

FP prepared the first draft of the manuscript. SR, BM and FP supervised the study in its clinical aspect. FP, SR and SS collected literature and supervised the background of the study. PC supervised the psychophysiological aspect of the study. CV created the web material. SS, PC and LM developed the first draft of the manuscript into the final version suitable for publication. AG, BW and GR conceived the idea of the study and supervised its scientific design. All authors read and approved the final manuscript.
